# Development and validation of a sensitive LC-MS/MS method for the quantitation of IMB-YH-4py5-2H, an antituberculosis candidate, and its application to the pharmacokinetic study

**DOI:** 10.1371/journal.pone.0228797

**Published:** 2020-02-19

**Authors:** Sen He, Hong-Tong Chen, Rui Zhao, Xin-Xin Hu, Tong-Ying Nie, Xin-Yi Yang, Cong-Ran Li, Xi Lu, Xiu-Kun Wang, Xue Li, Yun Lu, Guo-Qing Li, Jing Pang, Xue-Fu You

**Affiliations:** Beijing Key Laboratory of Antimicrobial Agents, Institute of Medicinal Biotechnology, Chinese Academy of Medical Sciences and Peking Union Medical College, Beijing, China; H Lee Moffitt Cancer Center and Research Institute, UNITED STATES

## Abstract

(E)-N,N-dimethyl-4-oxo-4-(4-(pyridin-4-yl)phenyl)but-2-enamide hydrochloride (IMB-YH-4py5-2H) is a novel Protein Kinase B (PknB) inhibitor with potent activity against *Mycobacterium tuberculosis* strains. In the present study, a sensitive and specific liquid chromatography/tandem mass spectrometry (LC-MS/MS) method was developed and validated to determine IMB-YH-4py5-2H in rat plasma. Sample pretreatment was achieved by liquid-liquid extraction with ethyl acetate, and separation was performed on an XTerra MS C_18_ column (2.1×50 mm, 3.5 μm) with gradient elution (methanol and 0.1% formic acid) at a flow rate of 0.3 mL/min. Detection was performed in multiple reaction monitoring (MRM) mode. Linear calibration curves were obtained over a concentration range of 1−100 ng/mL. The intra-day and inter-day precisions were lower than 8.46%, and the accuracies ranged from -8.71% to 12.36% at all quality control levels. The extraction recoveries were approximately 70%, and the matrix effects were negligible. All quality control samples were stable under different storage conditions. The validated method was successfully applied to a preclinical pharmacokinetic study in Sprague-Dawley rats. IMB-YH-4py5-2H demonstrated improved pharmacokinetic properties (higher exposure level) compared with its leading compound. IMB-YH-4py5-2H was also distributed throughout the lung pronouncedly, especially inside alveolar macrophages, indicating its effectiveness against lower respiratory infections.

## Introduction

Tuberculosis (TB) is an infectious disease caused by *Mycobacterium tuberculosis* that most often affects the lungs. As reported by the World Health Organization (WHO), there were an estimated 10.0 million new cases and 1.6 million deaths in 2017 [1], and TB has surpassed HIV/AIDS as the leading cause of mortality among infectious diseases. In the past two decades, marked progress has been made in reducing TB-related mortality worldwide. However, drug-resistant TB continues to be a public health crisis. The increasing prevalence of multidrug-resistant (MDR) and extensively drug-resistant (XDR) strains limit the application of marketed anti-TB drugs and represent a huge challenge for clinical practice [2,3]. Therefore, research into novel anti-TB targets and drug candidates is of paramount importance.

**Fig 1:**
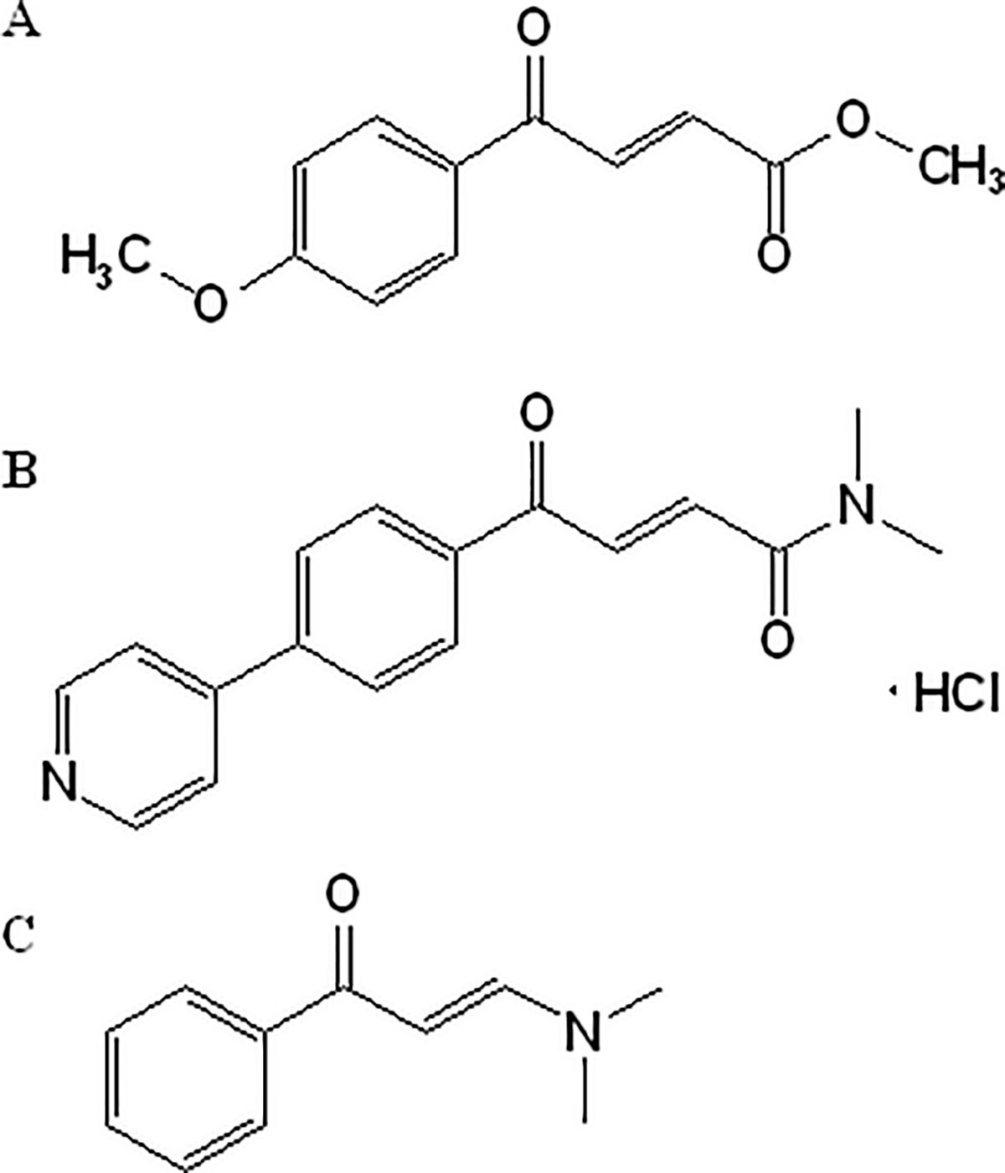
Chemical structures of YH-8 (A), IMB-YH-4py5-2H (B) and IS (C).

Protein kinase B (PknB), one of 11 serine/threonine protein kinases in *M*. *tuberculosis*, is essential for cell wall biosynthesis, cell division, and bacterial growth. The intracellular domain of PknB is the most active domain in the holoenzyme and is able to autophosphorylate and combine with ATP and its analogs [4–7]. Changes in the morphology and viability of *M*. *tuberculosis* can be caused by inhibiting the expression and phosphorylation of PknB [8–11]. PknB has a similarity of less than 30% to eukaryotic kinases, indicating that PknB is an attractive target for anti-TB drugs. A large number of PknB inhibitors with remarkable anti-TB capability, including aminopyrimidines, aminoguanidines and anthraquinones [12–17], have been reported.

(*E*)-Methyl-4-aryl-4-oxabut-2-enoate (YH-8, Fig 1) was identified as a PknB inhibitor suppressing both drug-sensitive and drug-resistant *M*. *tuberculosis* strains with significantly lower minimum inhibitory concentrations (MICs, 0.25–1 μg/mL) than other reported PknB inhibitors [18,19]. However, YH-8 was demonstrated to have unsatisfactory pharmacokinetic characteristics due to its poor solubility and low systemic exposure [20]. (E)-N,N-dimethyl-4-oxo-4-(4-(pyridin-4-yl)phenyl)but-2- enamide hydrochloride (IMB-YH-4py5-2H, Fig 1), a derivative of YH-8, was designed and synthesized. Potent activity against sensitive and resistant *M*. *tuberculosis* strains (MICs, 0.25–0.5 μg/mL) and improved water solubility make it a promising anti-TB candidate for further research.

Preclinical pharmacokinetic studies are helpful in predicting the *in vivo* behavior of a drug and providing increased knowledge for safe and efficient clinical applications. Hence, the present study aimed to establish and validate an accurate, sensitive and reliable liquid chromatography/tandem mass spectrometry (LC-MS/MS) assay for the routine analysis of IMB-YH-4py5-2H in rat plasma and to apply the assay to a preclinical pharmacokinetic study.

## Experimental

### Chemicals and reagents

IMB-YH-4py5-2H (purity>98%) was synthesized and purified by the Institute of Medicinal Biotechnology, Chinese Academy of Medical Sciences (Beijing, China). 3-(Dimethylamino)-1-phenyl-2-propen-1-one (Fig 1) was purchased from Sigma–Aldrich (Darmstadt, Germany) as an internal standard (IS, CAS: 1201-93-0, purity > 99%). HPLC-grade methanol and formic acid were purchased from Fisher Scientific (Fair Lawn, NJ, USA). Ultrapure water was obtained from a Millipore system (Bedford, MA, USA). All other reagents were of analytical grade. Pooled human liver microsomes, NADPH-regenerating system, and recombinant P450 enzymes (CYP1A2, CYP2A6, CYP2B6, CYP2C9, CYP2C19, CYP2D6, CYP2E1, CYP3A4 and CYP3A5) were purchased from Corning (Woburn, MA, USA).

### Instrumentation and condition

Separation was performed on a Shimadzu HT high-performance liquid chromatography (HPLC) system (Shimadzu, Japan) equipped with a binary pump (LC-20Adxr), an autosampler (SIL-20Acxr), and a column oven (CTO-20AC). The mobile phase consisting of a mixture of water (A) and methanol (B), both containing 0.1% formic acid, was delivered for separation by gradient elution at a flow rate of 0.3 mL/min. The gradient elution was programmed as follows: 0–2.0 min, 10% B; 2.0–3.0 min, 10%-60% B; 3.0–6.0 min, 60% B; 6.0–6.1 min, 60%-10% B; 6.1–8.0 min, 10% B. Five microliters of the processed samples were injected and separated on an XTerra MS C_18_ column (2.1×50 mm, 3.5 μm; Waters, Wexford, Ireland) maintained at 35°C.

Quantitation of IMB-YH-4py5-2H and the IS was achieved by MS/MS detection in positive ion mode using an AB SCIEX Qtrap 6500 mass spectrometer (Foster, CA, USA) equipped with an electronic spray ion (ESI) source at a temperature of 550°C and an ion spray voltage of 5500 V. Detection was performed in multiple reaction monitoring (MRM) mode by monitoring the transitions of m/z 281.3→210.1 (collision energy 40 eV) for IMB-YH-4py5-2H and m/z 176.9→104.9 (collision energy 22 eV) for IS. Data acquisition and processing were performed by Analyst (Version 1.6.2, AB SCIEX Instruments, Foster, CA, USA).

### Preparation of calibration standards and quality controls (QCs)

IMB-YH-4py5-2H and IS were accurately weighed into volumetric flasks and dissolved in an appropriate volume of methanol to prepare the stock solutions at a final concentration of 1 mg/mL. Working solutions of IMB-YH-4py5-2H were prepared by subsequent dilutions of stock solution with methanol. Seven-point calibration standards of 1, 2, 5, 10, 20, 50, and 100 ng/mL were prepared by spiking blank rat plasma (98 μL) with 2 μL of the IMB-YH-4py5-2H working solutions at appropriate concentrations. QCs were prepared by spiking blank plasma with IMB-YH-4py5-2H to the concentrations of 2.5 ng/mL (low quality control, LQC), 20 ng/mL (medium quality control, MQC) and 80 ng/mL (high quality control, HQC). All stock and working solutions were stored at −20°C.

### Sample processing

A simple liquid-liquid extraction method was employed for the extraction of IMB-YH-4py5-2H from rat plasma. A 100 μL aliquot of biological sample was spiked with 2 μL of IS working solution (500 ng/mL) and 2 μL of sodium chloride solution (250 mM). The mixture was extracted with 300 μL of ethyl acetate, vortexed for 1 min, and centrifuged at 6000 × *g* for 15 min. The supernatant was transferred into a new centrifuge tube. The extraction process was repeated one more time. Then, the combined supernatant was evaporated to dryness under a centrifugal vacuum evaporator (Christ Alpha 2–4 LD plus, Osterode am Harz, Germany). The residue was reconstituted with 100 μL of methanol containing 1% formic acid and centrifuged at 20000 × *g* for 5 min. Five microliters of the supernatant was injected into the LC-MS/MS system for analysis.

### Method validation

A full validation was performed for the assay according to the US Food and Drug Administration (USFDA) Bioanalytical Method Validation Guidance [21].

#### Selectivity

Selectivity was evaluated by comparing the chromatograms of six individual blank plasma samples with the corresponding standard-spiked plasma samples to ensure that there was no significant endogenous interference at the retention time of the analytes.

#### Linearity and sensitivity

The calibration curve was obtained by plotting the peak area ratios of IMB-YH-4py5-2H to the IS (y) against the nominal concentrations of IMB-YH-4py5-2H (x) at seven concentrations ranging from 1 to 100 ng/mL. The linearity was determined by least-squares linear regression with a weighting index of 1/x^2^. Lower limit of quantitation (LLOQ) was defined as the lowest concentration in the calibration curve with qualified precision (<20%) and accuracy (<±20%).

#### Precision and accuracy

The intra-day precision and accuracy were assessed by analyzing six replicates at three different QC levels (2.5, 20 and 80 ng/mL) in rat plasma. Inter-day accuracy and precision were evaluated by the analysis of QCs on three consecutive days. The precision and accuracy were expressed by the relative standard deviation (RSD% = (standard deviation/mean) × 100) and relative error (RE% = [(mean measured concentration − nominal concentration)/nominal concentration] × 100), respectively. The accuracies should be within ±15% at all QC levels, and the precisions should be no more than 15%.

#### Extraction recovery and matrix effect

Blank plasma from six different sources was used to assess the extraction recovery and matrix effect. Recovery was determined by comparing the peak area of IMB-4py5-2H from the QC sample extracted using the liquid-liquid extraction method with that from post-extracted spiked supernatant at the corresponding concentration. The matrix effect is generally due to the influence of co-eluting compounds on the ionization process of the actual analyte. The matrix effect was measured by comparing the peak area of IMB-4py5-2H in post-extracted spiked samples with that of a neat solution containing an equivalent amount of the compound.

#### Stability

Six replicates of the QC samples at low, medium and high concentrations subjected to the conditions below were evaluated for the stability of IMB-4py5-2H in rat plasma. Short-term stability was assessed by the analysis of the QCs kept at room temperature for 24 h, which exceeded the routine preparation time of the samples. Long-term stability was determined by assaying the QCs after storage for 2 weeks at −20°C. Freeze–thaw stability was investigated after three freeze (−20°C)–thaw (room temperature) cycles. Post-preparative stability was assessed by analyzing the extracted QC samples kept in autosampler at 4°C for 24 h.

### Animal experiment

Animal experiments were approved by the Ethics Committee of the Institute of Medicinal Biotechnology, Chinese Academy of Medical Sciences and Peking Union Medical College, with the approval number of IBM20170324D601. The research was conducted in accordance with the United States National Institutes of Health Guide for Care and Use of Laboratory animals. Adult Sprague-Dawley (SD) rats (weighing 190–210 g) were purchased from Beijing Vital River Laboratory Animal Technology Co., Ltd. (Beijing, China). The rats were housed under controlled environmental conditions (temperature of 22±2°C and humidity of 55±6%) with free access to standard laboratory food and water. All rats were acclimatized in the laboratory conditions for three days before the experiments and euthanized with CO_2_ after experiments.

#### Pharmacokinetic study in rats

A total of 18 adult SD rats were randomly divided into three dosing groups (half male and half female). IMB-YH-4py5-2H suspensions were freshly prepared in 1% (w/v) sodium carboxymethyl cellulose (CMC-Na). The rats were fasted with free access to water for 12 h prior to the oral administration of IMB-YH-4py5-2H at doses of 50, 100 or 200 mg/kg and fasted for another 2 h after administration. Blood samples of 0.2 mL were collected via the post-orbital venous plexus vein at 0.033, 0.083, 0.167, 0.333, 0.5, 0.75, 1, 1.5, 2, 4, 6, 8, 12, 24, 36, 48, and 60 h post dosing into a heparinized tube. Plasma samples, obtained by centrifuging blood at 2300 × *g* for 15 min at 4°C, were stored at −20°C until analysis.

#### Distribution of IMB-YH-4py5-2H in lung

After the oral administration of 100 mg/kg IMB-YH-4py5-2H, rats from each group (six rats per group and three rats per sex) were sacrificed at 5 min, 0.5 h, 8 h, and 30 h. Rat lungs were collected, rinsed with saline, wiped dry, accurately weighed and homogenized with ultrapure water. The homogenate was kept at −20°C until analysis.

As two representative infectious sites in lower respiratory tract infections, lung epithelial lining fluid (ELF) and alveolar macrophages (AMs) were investigated to determine the distribution of IMB-YH-4py5-2H. Freshly collected rat lungs were lavaged three times from the trachea with 5 mL of ice-cold phosphate buffer (pH 7.4). The lavage fluid was centrifuged at 4°C (650 × g, 5 min) to separate AMs from the ELF, immediately. The cell counts of the resuspended AMs were assessed by using the haemocytometer. The AMs were extracted with 1 mL of 0.1 M NaOH for analysis. To calculate the concentrations of IMB-YH-4py5-2H in ELF and AMs, reported data were employed. As reported, the total volume of ELF was estimated to be 393 μL/rat (~200 g body weight) [22–24], and the intracellular volume of a single AM was 1166 μm^3^ [25]. Based on the reported volume of a single AM, the raw data of IMB-YH-4py5-2H concentrations in AMs, expressed as ng/10^5^ cells, were converted to the values with the unit of μg/mL (Eq 1), which was consistent with the unit of concentrations in plasma and ELF and easier for comparison.

$$C_{AM\;(\mu g/mL)}=\frac{C_{AM\;(ng/10^{5}\;cells)}}{10^{5}\times V}\times 10^{9}$$ (1)

C_AM (μg/mL)_ is the concentration of IMB-YH-4py5-2H expressed as μg/mL, C_AM (ng/10_^5^_cells)_ is the concentration with the unit of ng/10^5^ cell, and *V* is the volume of a single AM (1166 μm^3^).

The homogenate, ELF, and AM samples were processed as described in “Sample processing” and injected into the LC-MS/MS system for analysis.

#### *In vitro* uptake study

Rat AMs NR8383 cells (American Type Culture Collection, Manassas, VA, USA) were grown overnight at 37°C with 5% CO_2_ in Ham’s F12K medium (Gibco, Grand island, NY, USA) containing 20% fetal bovine serum (FBS, Gibco, Thornton, Australia) in 24-well culture plates (4 × 10^5^ cells/well in 1 mL culture medium). After 24 h-exposure to IMB-YH-4py5-2H (2 μg/mL), the culture medium and the subsequent three washes were pooled and processed further for the determination of the extracellular drug concentrations. The cells were then extracted with 0.1 M NaOH to collect the intracellular drug for the LC-MS/MS analysis. All experiments were done in triplicate. The intracellular/extracellular drug concentration ratios were then calculated.

#### Intracellular activity assay

In order to confirm that the high concentration of IMB-YH-4py5-2H in AMs contributed to the anti-TB effect, we performed its inhibition study on intracellular *M*. *tuberculosis*.

Mouse AMs J744 cells (4 × 10^5^ cells/well in 1 mL culture medium) were grown overnight in DMEM medium (Gibco, Grand island, NY, USA) containing 10% FBS in 24-well plates. *M*. *tuberculosis* H37Rv cultures were diluted to infect macrophages at a multiplicity of infection of 10 bacteria per cell. After a 4 h-incubation, the cells were washed three times with fresh media to remove the extracellular *mycobacteria*. The medium with different concentrations of IMB-YH-4py5-2H (0.5, 1, and 2 μg/mL) was replaced daily. After a three-day incubation, the medium was removed and the macrophages were lysed with 200 μL 0.1% sodium dodecyl sulfate. The lysates were diluted with fresh media and plated onto 7H10 plates supplemented with 10% OADC enrichment to measure the colony forming units (CFU) of the bacteria.

### Metabolic stability assay

IMB-YH-4py5-2H was incubated at 37°C for 0, 15, 30, 45, and 60 min in human liver microsomes (HLMs) for the metabolic stability study. The incubation system included 0.1 mg/mL pooled liver microsomes, 100 mM phosphate-buffered saline (PBS, pH 7.4), NADPH regeneration system (the final concentration contained 3.3 mM glucose-6-phosphate, 1.3 mM NADP^+^, 0.4 U/mL glucose-6-phosphate dehydrogenase and 3.3 mM MgCl_2_), and 1 μM IMB-YH-4py5-2H.

In addition, to characterize the enzymes that may be involved in microsomal metabolism, IMB-YH-4py5-2H was incubated at 37°C for 30 min with different recombinant human P450 enzymes, i.e., CYP1A2, CYP2A6, CYP2B6, CYP2C8, CYP2C9, CYP2C19, CYP2D6, CYP2E1, CYP3A4 and CYP3A5. The incubation system included CYP isoenzyme at a final concentration of 0.05 nmol/mL, 100 mM PBS (pH 7.4) or 100 mmol/L Tris buffer (pH 7.5, Tris buffer was used in the CYP2C9 and CYP2A6 reaction systems and PBS was used for the other enzymes), NADPH regeneration system, and 1 μM IMB-YH-4py5-2H.

After incubation, the samples were processed as described in “Sample processing”. All experiments were performed in triplicate. The concentration of IMB-YH-4py5-2H was measured using LC-MS/MS analysis.

### Data analysis

The pharmacokinetic parameters of IMB-YH-4py5-2H were calculated by WinNonlin (Version 6.1, Pharsight Corp., Mountain View, CA, USA) using a non-compartmental model. Data were expressed as the mean ± standard deviation (S.D.).

The inhibitory effects of different IMB-YH-4py5-2H concentrations against intracellular *M*. *tuberculosis* H37Rv were evaluated by one-way ANOVA employing SPSS 16.0. Differences were considered statistically significant at *P* < 0.05.

## Results and discussion

The essential role of PknB in sustaining the growth of *M*. *tuberculosis* makes it a potential target for developing TB inhibitors. Compound YH-8, synthesized previously, is a novel inhibitor of PknB and potently prevents the growth of *M*. *tuberculosis in vitro*. However, the pharmacokinetic study performed by Zhai et al. revealed that serum *C*_max_ values of YH-8 following oral administration of 200 mg/kg were lower than 0.5 μg/mL and decreased below the therapeutic range in less than 1 hour [20]. The poor pharmacokinetic profiles of YH-8 hindered its further development. IMB-YH-4py5-2H, a derivative of YH-8, showed better water solubility and comparable activity (MICs between 0.25–0.5 μg/mL). For the purpose of gaining additional knowledge about the preclinical pharmacokinetic characteristics of IMB-YH-4py5-2H, a sensitive and specific analytical method should be developed first.

### Method development

Mobile phases and commercially available HPLC columns were optimized to improve the chromatographic behavior and ionization response of IMB-YH-4py5-2H. The mobile phase consisting of methanol and water contributed the most intense response, and the gradient elution on a Waters XTerra MS C18 column achieved satisfactory chromatographic separation with a negligible matrix effect. Liquid-liquid extraction was employed as the sample preparation method due to the cleaner background and higher extraction efficiency achieved with this method than with protein precipitation. The sodium chloride added to the biological sample during the sample processing can increase the extraction recovery further to approximately 70%.

### Analytical method validation

A full validation, including specificity, linearity, precision, accuracy, extraction recovery, matrix effect, and stability, was conducted in rat plasma according to the USFDA guidelines. All of the parameters were proven to meet the criteria for bioanalytical method validation.

#### Selectivity

**Fig 2:**
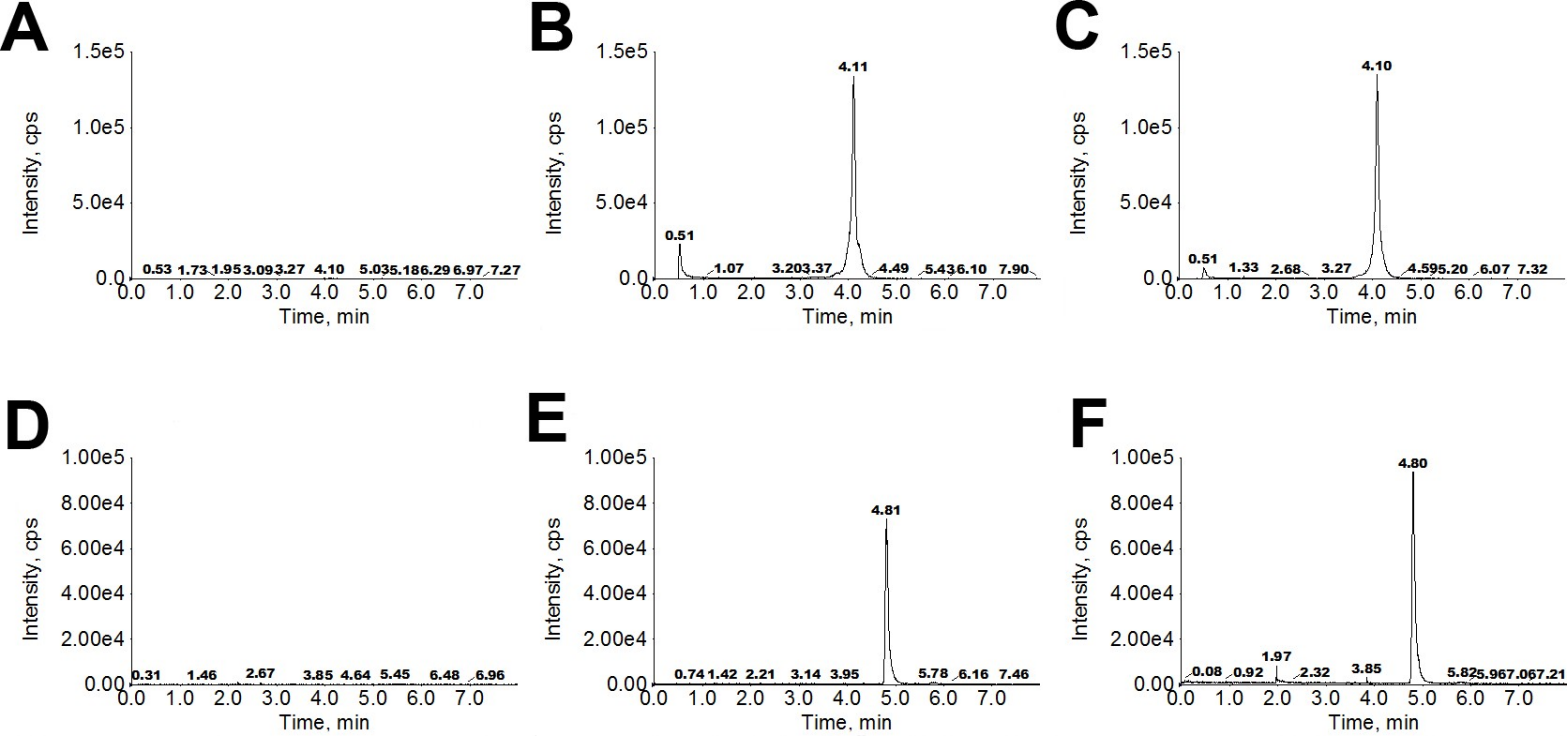
Representative LC-MS/MS chromatograms of IMB-YH-4py5-2H (upper row) and the IS (bottom row). Blank rat plasma (A, D); blank plasma spiked with 100 ng/mL IMB-YH-4py5-2H and 10 ng/mL IS (B, E); plasma sample collected 0.5 h following the oral administration of 100 mg/kg IMB-YH-4py5-2H (C, F).

Typical chromatograms of blank rat plasma, blank plasma spiked with IMB-YH-4py5-2H (100 ng/mL) and IS (10 ng/mL), and plasma samples collected 0.5 h after oral administration are shown in Fig 2. Compared with the spiked plasma samples, all blank plasma samples from six individual rats were found to be free from interference of endogenous substances at the retention time of IMB-YH-4py5-2H and the IS in the MRM scan mode. The chromatographic behavior of the real plasma samples was similar to that of the spiked plasma samples.

#### Calibration curve and lower limit of quantification

The calibration curve exhibited good linearity over the concentration range of 1–100 ng/mL. The typical standard curve was described by the equation y = 1530x+55 (*R*^*2*^ = 0.999), where y is the peak area ratio and x is the concentration of IMB-YH-4py5-2H. The deviation of the calculated concentration from the nominal concentration was within the acceptable criteria of 15%. The LLOQ was 1 ng/mL with qualified accuracy (<±20%) and precision (<20%).

#### Precision and accuracy

As presented in Table 1, the intra- and inter-day precisions of all three QC levels were less than 8.46%, and the accuracies were between -8.71% and 12.36%. The precision and accuracy meet the criteria of bioanalytical method validation according to USFDA guidance [21].

**Table 1:** Precision and accuracy of IMB-YH-4py5-2H in rat plasma.

Nominal	Intra-day		Inter-day	
concentration	Accuracy	Precision	Accuracy	Precision
(ng/mL)	(%, n = 6)	(RSD%, n = 6)	(%, n = 18)	(RSD%, n = 18)
2.5	12.36	8.46	6.47	6.51
20	-6.58	4.76	-1.35	7.49
80	-8.71	7.37	-8.51	7.07

#### Extraction recovery and matrix effect

The extraction recoveries of IMB-YH-4py5-2H from rat plasma were 71.35 ±3.49%, 70.30 ±5.81%, and 69.99 ±2.98% at the concentrations of 2.5, 20, and 80 ng/mL, respectively. The matrix effects of IMB-YH-4py5-2H for all QC levels were between 85.0 and 115.0%, which demonstrates no significant matrix effect with this method.

#### Stability

The results of all the stability studies are summarized in Table 2. IMB-YH-4py5-2H was found to be stable at room temperature for 24 h, at −20°C for 2 weeks, after three freeze–thaw cycles, and after storage in the autosampler at 4°C for 24 h. IMB-YH-4py5-2H was proven to be stable throughout the entire process of the analysis.

**Table 2:** Stability data of IMB-YH-4py5-2H in rat plasma (n = 6).

Nominal concentration	%Theoretical			
(ng/mL)	24 h ambient	Long-term	freeze-thaw	Autosampler
2.5	94.35 ± 3.65	92.33 ± 4.21	90.11 ± 6.19	100.11 ± 1.20
20	91.96± 5.79	91.65 ± 3.91	88.79 ± 3.27	103.26 ± 6.07
80	103.77 ±2.51	94.98 ± 7.26	94.17 ± 5.10	97.85 ± 4.96

### Pharmacokinetic study of IMB-YH-4py5-2H

**Fig 3:**
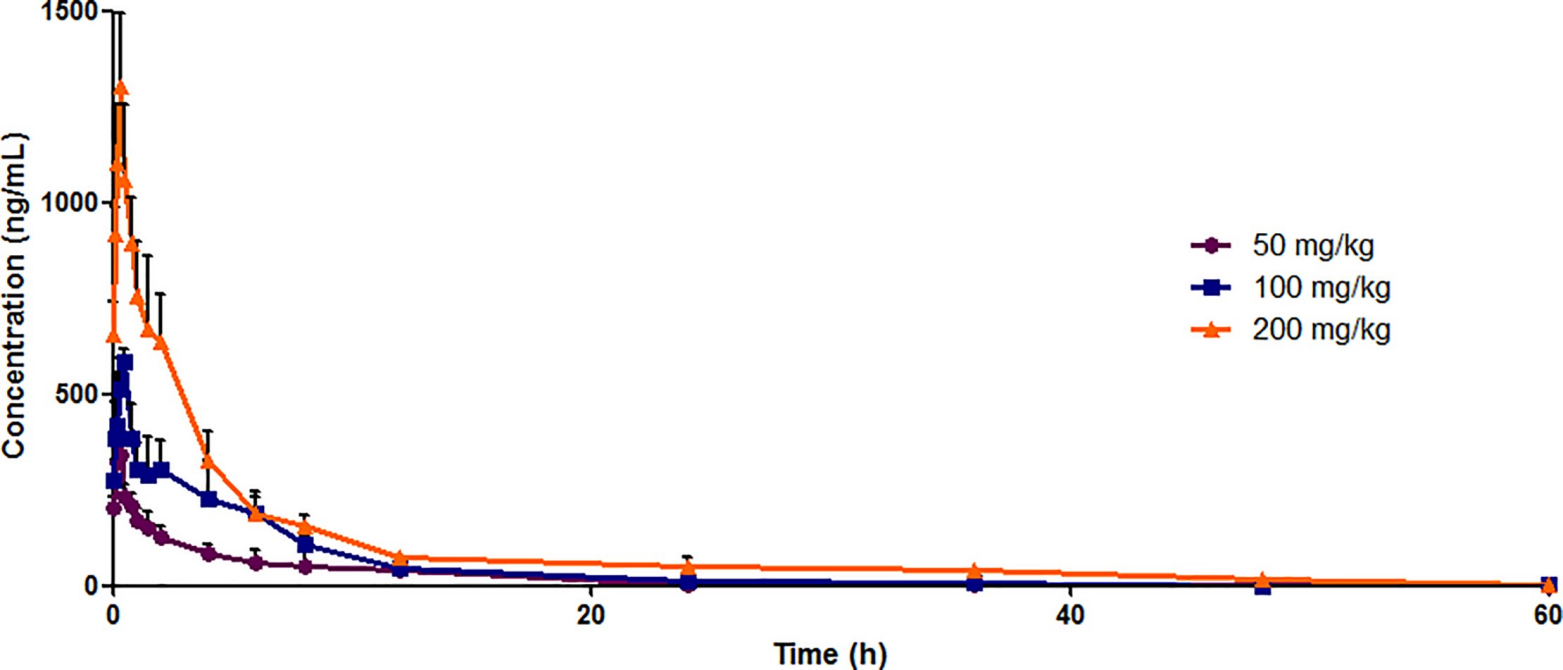
Comparison of the mean plasma concentration−time curves following different oral doses of IMB-YH-4py5-2H. Each point represents the mean ± S.D. (n = 6).

The developed method was successfully applied to the pharmacokinetic study of IMB-YH-4py5-2H in SD rats following a single oral administration of 50, 100 and 200 mg/kg. The bioanalytical method was sensitive enough for the quantification of IMB-YH-4py5-2H in rat plasma up to 60 h, as shown in the mean plasma concentration−time curves (Fig 3). The pharmacokinetic parameters of IMB-YH-4py5-2H calculated by Phoenix WinNonlin using a non-compartmental model are listed in Table 3. The time to maximum concentration (*T*_max_) of 0.28±0.08, 0.44±0.09, and 0.33±0.00 h for the 50, 100, and 200 mg/kg dosing groups, respectively, revealed that IMB-YH-4py5-2H was rapidly absorbed. IMB-YH-4py5-2H was gradually eliminated with a half-life of approximately 7 h for all three dosing groups. Both the peak plasma levels (*C*_max_) and areas under the plasma concentration-time curve (AUC) of IMB-YH-4py5-2H increased in a dose-dependent manner. The *C*_max_ of IMB-YH-4py5-2H after an oral dose of 100 and 200 mg/kg was 586.33±31.25 and 1302.83±193.89 ng/mL, respectively, which is above the *in vitro* effective concentration (MIC) of 0.25–0.5 μg/mL, implying that effective therapeutic levels may be achievable *in vivo*. The AUC of IMB-YH-4py5-2H was approximately 2.5-fold that of YH-8 following the same oral dose, which indicated better *in vivo* efficacy [20].

**Table 3:** Pharmacokinetic parameters of IMB-YH-4py5-2H following a single oral dose of 50, 100, and 200 mg/kg in rats (n = 6).

Pharmacokinetics parameters	50 mg/kg	100 mg/kg	200 mg/kg
*T*_max_ (h)	0.28±0.08	0.44±0.09	0.33±0.00
*C*_max_ (ng/mL)	366.67±23.01	586.33±31.25	1302.83±193.89
AUC_0-60_ (h•ng/mL)	1402.29±367.43	2835.76±424.59	5711.18±659.14
AUC_0-∞_(h•ng/mL)	1437.91±370.07	2848.51±429.98	5728.45±663.53
*t*_1/2_ (h)	7.24±1.61	6.37±0.93	7.71±0.41
*Vz/F* (L/kg)	378.59±100.19	336.49±74.02	393.93±58.05
*CL/F* (L/h/kg)	36.77±9.68	36.60±6.13	35.34±4.45
MRT (h)	7.63±1.58	7.70±1.43	10.72±0.85

The concentrations of IMB-YH-4py5-2H in plasma, lung homogenate, ELF and AMs after oral administration are shown in Table 4. The concentration of an antibacterial agent at the infected tissue is generally considered to be a more important factor for therapeutic effects than the concentration in plasma. The response of intracellular bacterial infections (e.g., pulmonary tuberculosis) may be predicted better with tissue to serum ratios, especially for infected body sites with a permeable membrane such as the alveolar epithelium [26]. The concentrations of IMB-YH-4py5-2H in lung homogenate were higher than those in plasma in the absorption and distribution phase. The high intrapulmonary drug concentrations observed in this study suggested satisfying effectiveness of IMB-YH-4py5-2H in the treatment of pulmonary tuberculosis.

**Table 4:** Concentrations of IMB-YH-4py5-2H following a single oral dose of 100 mg/kg in rat plasma, lung homogenate, ELF, and AMs (n = 6).

	Concentration				
Time	Plasma	lung homogenate	ELF	AMs	
(h)	(μg/mL)	(μg/g)	(μg/mL)	(ng/10^5^ cells)	(μg/mL)
0.083	0.917±0.071	4.407±0.607	0.639±0.093	166.763±40.110	1494.295±359.408
0.5	1.062±0.192	10.852±1.960	1.804±0.264	227.198±35.589	2035.827±318.898
8	0.155±0.029	0.107±0.022	0.121±0.032	11.128±4.099	99.716±36.729
30	0.039±0.012	0.011±0.003	0.050±0.045	2.823±2.164	25.299±19.391

ELF and AMs, representing extracellular and intracellular sites, respectively, have been increasingly studied as predictors of drug behaviors in pulmonary infections. We investigated the distribution of IMB-YH-4py5-2H in ELF and AMs *in vivo* after oral administration to rats and revealed that IMB-YH-4py5-2H was efficiently distributed in ELF and AMs. The concentrations of IMB-YH-4py5-2H in ELF were comparable to that in plasma. Notably, IMB-YH-4py5-2H was highly concentrated in AMs, and the IMB-YH-4py5-2H concentrations in AMs were significantly higher than the MIC until 30 h. This pronounced tissue distribution was considered contributing to good *in vivo* antibacterial efficacy of IMB-YH-4py5-2H in the treatment of lower respiratory infections, especially to the intracellular organisms.

The *in vitro* uptake study further demonstrates an extraordinary accumulation of IMB-YH-4py5-2H in AMs. The intracellular to extracellular concentration ratio (I/E ratio) was 133.4±12.5 after 24 h-exposure in 2 μg/mL of IMB-YH-4py5-2H.

High accumulations of the antibacterial agents in AMs, including rifampicin and azithromycin, were reported in several other publications [27, 28]. Besides, lipophilic drugs were reported to show higher intracellular accumulation in AMs *in vitro* [28, 29]. Therefore, one possible explanation for the significant accumulation of IMB-YH-4py5-2H in AMs is its relatively high hydrophobicity.

The activity of IMB-YH-4py5-2H against *M*. *tuberculosis* inside macrophages was also evaluated. IMB-YH-4py5-2H produced a 0.45 log_10_CFU (approximate 70% bacterial density) reduction on intracellular *M*. *tuberculosis* at the extracellular concentration of 2 μg/mL (S1 Table). These results further demonstrate that IMB-YH-4py5-2H can target the intracellular pathogens. Besides, the leading compound YH-8 was also reported to exhibit anti-TB activity against both cell-free and intracellular *mycobacteria* [19].

**Fig 4:**
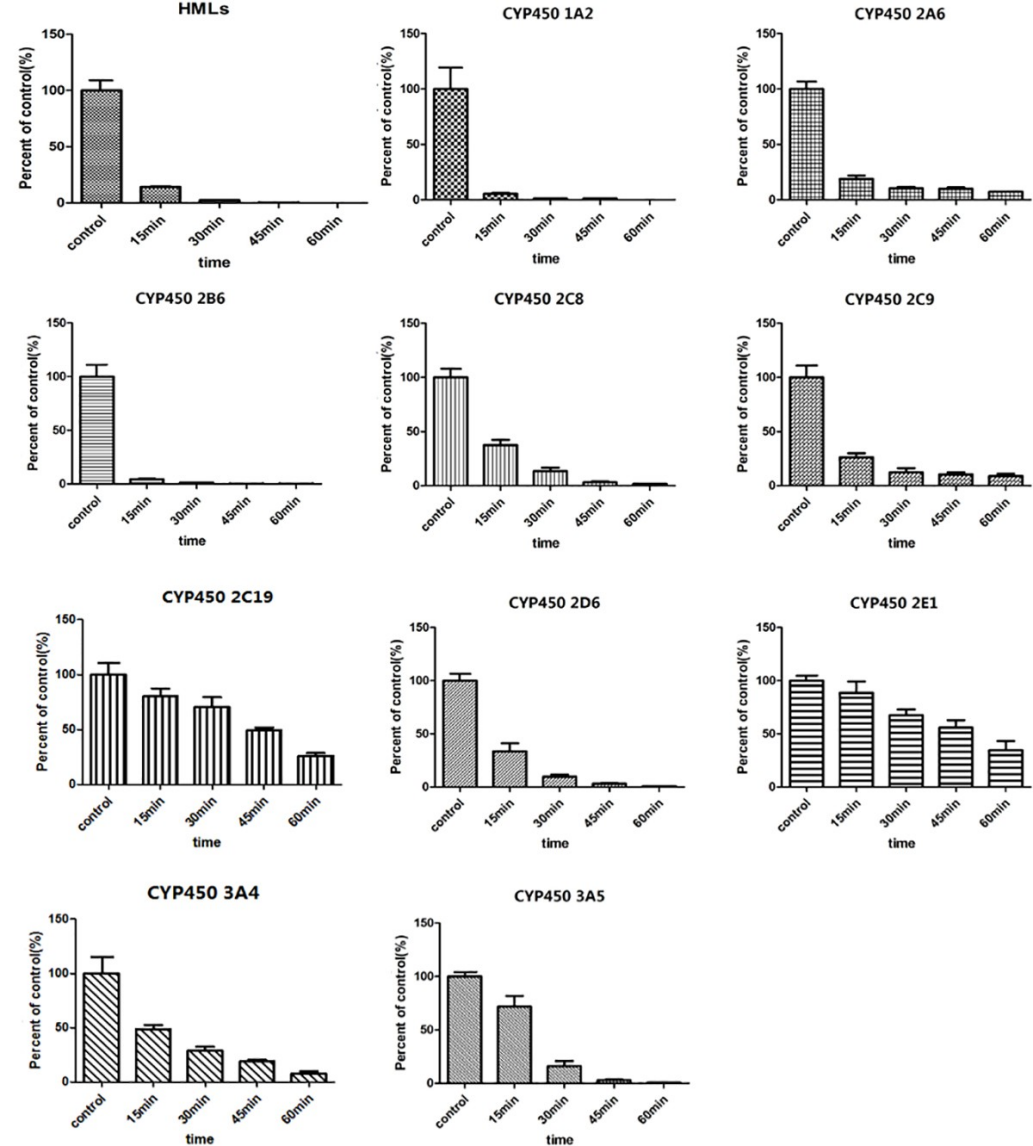
Metabolism of IMB-YH-4py5-2H in HLMs and with each CYP enzyme (n = 3).

To better understand the pharmacokinetic behavior of IMB-YH-4py5-2H, its metabolic stability in HLMs was investigated. IMB-YH-4py5-2H was rapidly metabolized in liver microsomes with a half-life of less than 15 min at 1 μM, as shown in Fig 4. Based on this finding, hepatic metabolism may play an important role in drug biotransformation. To characterize the enzymes that may be involved in the microsomal metabolism of IMB-YH-4py5-2H, IMB-YH-4py5-2H was incubated with human recombinant CYP supersomes. Fig 4 shows that all the CYP isozymes tested may be involved in the metabolism of IMB-YH-4py5-2H. In particular, CYP1A2, CYP2A6, and CYP2B6 showed high metabolic activity.

## Conclusions

A sensitive and specific LC-MS/MS method for the quantification of IMB-YH-4py5-2H in rat plasma was developed, validated, and applied to the pharmacokinetic study of IMB-YH-4py5-2H. Increased knowledge about the preclinical pharmacokinetic characteristics of IMB-YH-4py5-2H was gained based on our study. A higher exposure level was identified for IMB-YH-4py5-2H than its leading compound YH-8. IMB-YH-4py5-2H also exhibited outstanding distribution capacity in the lung, especially inside alveolar macrophages, which indicated promising efficacy against lower respiratory infections. The information we obtained will be meritorious for further research, particularly for the design of dosage regimen.

## Supporting information

S1 Table Activity of IMB-YH-4py5-2H against intracellular *M*. *tuberculosis* H37Rv. (DOCX)

S1 File The ARRIVE guidelines checklist. (PDF)
