# Supplementary material for: Development and validation of a sensitive LC-MS/MS method for the quantitation of IMB-YH-4py5-2H, an antituberculosis candidate, and its application to the pharmacokinetic study
Source: PLoS One. 2020 Feb 19;15(2):e0228797. doi: 10.1371/journal.pone.0228797 (PMC7029871; doi:10.1371/journal.pone.0228797)
Supplement: S1 Table — (DOCX) [file pone.0228797.s001.docx]

**S1 Table** **Activity of IMB-YH-4py5-2H against intracellular *M. tuberculosis* H37Rv. Data represents the mean ± S.D. (n = 3).**

| Treatment | Log_10_CFU | | | |
| --- | --- | --- | --- | --- |
|  | 2 μg/mL | 1 μg/mL | 0.5 μg/mL | Untreated |
| IMB-YH-4py5-2H | 6.02±0.11* | 6.40±0.05 | 6.44±0.02 | 6.47±0.04 |
| Isoniazid | 4.01±0.14* | 3.57±0.06* | 4.60±0.05* |  |

**P* <0.05 compared to the untreated group.
